# Parental correlations of physical activity and body mass index in young children- the GECKO Drenthe cohort

**DOI:** 10.1186/s12966-015-0295-0

**Published:** 2015-10-09

**Authors:** Anna Sijtsma, Pieter JJ Sauer, Eva Corpeleijn

**Affiliations:** Department of Epidemiology (FA40), University Medical Center Groningen, University of Groningen, P.O. Box 30.001, 9700 RG Groningen, Netherlands; Department of Pediatrics (CA70), University Medical Center Groningen, University of Groningen, P.O. Box 30.001, 9700 RG Groningen, Netherlands

**Keywords:** Young children, Preschool, Waist circumference, Commuting, Sport, Offspring, Accelerometer, Tracmor

## Abstract

**Background:**

Parental behavior can influence the development of overweight in children. The aim of this study is to examine whether parental BMI and parental physical activity are associated with BMI, waist circumference and physical activity in young children.

**Methods:**

In 3–4 year old children, weight, height and waist circumference were measured. Children’s physical activity was measured in a subgroup (*n* = 299) using a tri-axial activity monitor, Tracmor_D_. Data are represented as activity counts per minute (total physical activity) and as percentage of time in sedentary, light, moderate and vigorous intensity physical activity (generated from a subsample of Actigraph data using cut points from Butte et al.). Parental weight and height were self-reported and parental physical activity was assessed by the validated questionnaire SQUASH.

**Results:**

In total 1554 children (age 3.9 ± 0.1 years, BMI 15.8 ± 1.3 kg/m2 and waist circumference 52.4 ± 3.5 cm) were included. Eleven percent were overweight or obese. A higher maternal BMI was related to higher levels of children’s sedentary activity (*r* = 0.120, *p* = 0.04 and to lower levels of children’s total and moderate physical activity (*r* = −0.158, *p* = 0.007 and *r* = −0.154, *p* = 0.008, respectively). Parental BMI was positively correlated with children’s BMI and waist circumference (*r* = 0.20–0.27, *p* < 0.001). Higher maternal total physical activity levels were not related to children’s total physical activity level, but were related to higher levels of children’s moderate and vigorous physical activity (ρ = 0.132, *p* = 0.046 and ρ = 0.132, *p* = 0.046, respectively). No correlations between total, moderate or vigorous physical activity levels of the parents with the child’s BMI or waist circumference were found. Looking at physical activity domains maternal physical activity in active commuting, either walking or biking, showed a negative correlation with BMI of the child (ρ = −0.062, *p* = 0.042).

**Conclusions:**

Higher maternal BMI and lower maternal physical activity levels were related to lower levels of children’s physical activity. More active commuting by the mother and a lower parental BMI were related to a lower BMI of the children. Energy-balance related behavior of the parents may contribute to a healthier BMI of both preschool children and their parents.

## Background

Childhood obesity increases the chance of obesity and obesity-related adverse health effects at adult age [1, 2], since overweight is hard to treat once it is established. Therefore, prevention of overweight in early life is important. Previous studies have shown that intervention at early age gives a high chance of success to both prevention and treatment of overweight or obesity in later life [3, 4].

Physical activity is an important energy-balance related behavior to prevent overweight, because it increases energy expenditure. Physical activity in early life might have health benefits later on, particularly with respect to body composition [5]. Habitual physical activity is associated with a lower percentage body fat in preschool children [6] and can protect children from developing overweight [7]. Besides prevention of overweight, physical activity has many other health benefits, like a better psychosocial health, cognitive development, cardiometabolic health, skeletal health and motor skill development [5]. Furthermore, the development of motor skills may play a role in participation in various forms of physical activity later in life [8]. Finally there is evidence that obesity [1], inactivity patterns [9] and physical activity levels [10] may all track from childhood to adolescence and adulthood.

Parents can influence the development of their children’s overweight at different phases of development. Already before and during pregnancy, high pre-pregnancy body mass index (BMI) and weight gain during pregnancy, can increase the risk for obesity in the offspring [11]. Even a higher level of physical activity during pregnancy may be related to a lower percentage of body fat at age 5 [12]. During infancy and early childhood parents play an important role in the development of habits related to diet and physical activity [13]. Parental physical activity and support of children’s physical activity might encourage children to be active in their daily habits, by being a role model for their children, or by actively participating in physical activity together with their children. However, studies investigating the effect of parental physical activity on the physical activity level of preschool children are limited.

The aim of our study is to examine whether parental BMI and parental physical activity are associated with BMI, waist circumference and physical activity in 3- and 4-year-old children.

## Methods

### Participants

This study is part of the Groningen Expert Center of Kids with Obesity (GECKO) Drenthe birth cohort. The GECKO Drenthe cohort is a population-based birth cohort studying early risk factors for overweight and obesity in young children. Children are born between 2006 and 2008 in Drenthe, a northern province of the Netherlands. Details of the study design, recruitment and study procedures were described in detail elsewhere [14]. For the present analyses, all children with available data were included. At baseline, in 2006–2008, parents of 2997 children intended to participate in the study, of whom 2874 actively participated. Missing data are mainly due to logistic and organizational problems. For all children, written informed consent was obtained from parents, and the study was approved by the Medical Ethics Committee of the University Medical Center Groningen (UMCG) and follows the declaration of Helsinki.

### Anthropometric measurements

Anthropometry in children was measured by trained nurses from Youth Health Care according to a standardized protocol. Weight was measured in light clothing using an electronic scale with digital reading, and recorded to the nearest 0.1 kg. Height was assessed using a stadiometer and recorded to the nearest 0.1 cm. Waist circumference was measured twice using a measure tape midway between the lowest rib and the top of the iliac crest at gentle expiration over the naked skin in standing position to the nearest 0.1 cm. When the two measurements differed more than 1 cm a third measurement was done. The mean of two close measurements was taken. BMI was calculated as weight (kg) divided by squared height (m). BMI-for-age Z-scores and waist circumference-for-age Z-scores were calculated according to the reference tables of the Netherlands 1997 [15] using Growth Analyser 3.5 © (Dutch Growth Foundation, Rotterdam, the Netherlands, http://www.growthanalyser.org). Overweight and obesity were defined according to Cole et al. (2000) [16]. BMI and waist circumference measurements were available at multiple time points (24, 36, 45 and 60 months of age) and the measurement most close to the age of physical activity assessment was chosen for analysis. Only those children with <0.1 years difference between the physical activity assessment and anthropometry were included.

Parental BMI was assessed from self-reported weight and height. Height (cm) was reported before birth of the children. Weight (kg) was reported before pregnancy (retrospectively) and at the children’s age of 18 months. Most recent available weight was used to calculate BMI. In 54 % of the mothers and 53 % of the fathers, BMI at child’s age 18 months was available. For the remaining mothers and fathers, pre-pregnancy weight was used. BMI values from those parents who had weight reported at both time points were highly correlated (mothers: *r* = 0.92, *p* < 0.001; fathers *r* = 0.89, *p* < 0.001). Maternal BMI at the two time points were comparable (mean difference = 0.5 ± 1.76, *p* = 0.263).

### Physical activity

Between 2 and 5 years of age, physical activity was assessed in a subset of children by the DirectLife tri-axial accelerometer, Tracmor_D_ (Philips DirectLife, Amsterdam, the Netherlands). Tracmor_D_ provides moderate-to-strong validity evidence that supports its use to evaluate physical activity levels and energy used for activity in 3- and 4-year-old children [17]. The accelerometer was worn when the children were awake, except for water activities, in a small case on a belt in the middle of the lower back. Output of the accelerometer is defined as mean activity counts per minute (ACM). This unit of measurement has been used previously in research with young children [18]. Data of the activity monitor is presented as the sum of all counts per minute when the monitor was worn divided by the total amount of minutes the monitor was worn. Sleeping time, including daytime sleeping, were recorded in a diary and not included in the analyses. To be included in the analysis, the accelerometer had to be worn at least 400 min/day for at least 3 days, including at least 1 weekend day and 2 week days [19]. No validated cut off points to determine percentage of time spent in sedentary, light, moderate and vigorous activity are available for the Tracmor_D._ Fourteen children in our study have worn both the Tracmor_D_ and the Actigraph GT3X accelerometer at the same time. Percentages of time spent in sedentary, light, moderate and vigorous activity according to the Actigraph cut points from Butte et al. [20]. were compared to the activity data from the Tracmor_D._ Based on these results we set the following cut points for the amount of counts in one minute: sedentary 0–1999; light activity 2000–9999; moderate activity 10 000 – 16 999; and vigorous activity 17 000 or more activity counts in one minute.

Parental physical activity was assessed by the validated questionnaire SQUASH (Short QUestionnaire to ASsess Health enhancing physical activity) [21]. The SQUASH estimates habitual physical activities and is structured in commuting activities, leisure time activities, sports, household activities, and activities at work or school. Questions included type of activity, duration, frequency and intensity. Total amount of physical activity in minutes per week was calculated and outcomes were analyzed as time spent in light physical activity, moderate physical activity and vigorous physical activity, as well as physical activity in categories of commuting, leisure time, sports, household, and activities at work or school. Parents who spent more than 7560 min/week (more than 18 h/day) on physical activity were excluded, because of their impossible high time in physical activity.

### Statistics

All analyses were done separately for maternal and paternal determinants. Data are presented as means ± SD or, if skewed, as medians with the 25^th^ to 75^th^ percentiles. For normally distributed variables, Pearson correlation coefficients (r) were used. For non-normal distributed variables, Spearman correlation coefficients (ρ) were used. Actively commuting mothers were compared non-active commuting mothers by t-test. Statistical analyses were performed using SPSS 20.0.0.1 for Windows (SPSS, Chicago Illinois, USA). The significance level was set to *p* < 0.05 (2-tailed).

## Results

Characteristics of all children together as well as for the subgroup that also had physical activity assessments are shown in Table 1. BMI was available for 1554 children. Children were on average 3.9 ± 0.1 years of age, had a BMI of 15.8 ± 1.3 kg/m^2^ and a waist circumference of 52.4 ± 3.5 cm. Eleven percent of the children were either overweight (8.4 %) or obese (2.3 %), as expected. Physical activity was measured in 299 children. Based on the criterion that the physical activity measurement and anthropometry in the children were less than 0.1 year apart, BMI was missing in 106 children and waist circumference was missing in 140 children. Characteristics of the parents are shown in Table 2.

**Table 1:** Characteristics of the children

	All children		Children with PA assessment	
	n	Mean ± SD or n (%)	n	Mean ± SD or n (%)
Boys	1554	780 (50 %)	299	157 (53 %)
Age (yrs)	1554	3.9 ± 0.1	299	3.7 ± 0.6
BMI (kg/m^2^)	1554	15.8 ± 1.3	298	15.9 ± 1.3
^a^OW/obese	1554	167 (11 %)	298	30 (10 %)
^b^BMI Z-score	1554	0.11 ± 0.92	298	0.09 ± 0.89
Waist (cm)	1164	52.4 ± 3.5	242	51.5 ± 3.6
^b^Waist Z-score	1164	0.39 ± 0.91	242	0.28 ± 0.93
^c^Total PA(ACM)	-	-	299	3715 ± 712
Sedentary activity (%)	-	-	299	47.1 ± 8.2
Light activity (%)	-	-	299	45.0 ± 7.4
Moderate activity (%)	-	-	299	6.0 ± 2.4
Vigorous activity (%)	-	-	299	1.9 ± 1.3

*ACM* counts per minute, *OW* overweight, *PA* physical activity, *SD* standard deviation, *yrs* years

^a^Overweight and obesity defined according to Cole 2000 [16]

^b^According to reference tables of the Netherlands, 1997

^c^Average counts per minute for wake hours

**Table 2:** Characteristics of the parents

	n	Mean ± SD or n (%)	Median [25^th^-75^th^ percentile]
Mother characteristics			
Age at birth child (yrs)	1480	31.4 ± 4.3	31.4 [28.6–34.5]
BMI (kg/m^2^)	1448	24.8 ± 4.7	23.8 [21.6–26.8]
^a^Overweight	1448	368 (24 %)	-
^b^Obese	1448	179 (12 %)	-
Total PA (min/week)	1079	3675 ± 1807	3330 [2580–4373]
LPA (min/week)	1079	2843 ± 1503	2610 [1860–3540]
MPA (min/week)	1079	767 ± 866	480 [240–1020]
VPA (min/week)	1079	66 ± 123	0 [0–90]
Father characteristics			
Age at birth child (yrs)	1439	34.2 ± 4.8	34.1 [31.1–37.0]
BMI (kg/m^2^)	1405	25.4 ± 3.4	25.0 [23.1–27.1]
^a^Overweight	1405	573 (37 %)	-
^b^Obese	1405	115 (7 %)	-
Total PA (min/week)	1023	3595 ± 1564	3420 [2880–1064]
LPA (min/week)	1023	2385 ± 1446	2610 [1170–3240]
MPA (min/week)	1023	1073 ± 1264	420 [150–420]
VPA (min/week)	1023	137 ± 234	0 [0–90]

*LPA* light physical activity, *min* minutes, *MPA* moderate physical activity, *PA* physical activity, *SD* standard deviation, *VPA* vigorous physical activity, *yrs* years

^a^BMI = 25.0–29.99

^b^BMI ≥ 30

Correlations between parental physical activity and BMI with children’s physical activity, BMI and waist circumference are shown in Tables 3 and 4. A higher BMI in both mother and father strongly correlated with a higher BMI Z-score and waist circumference Z- score of the children. A higher maternal BMI was related to lower levels of children’s total physical activity (*r* = −0.158, *p* = 0.007). After stratification for sex this was stronger in girls (*r* = −0.167, *p* = 0.04) than in boys (*r* = −0.133, *p* = 0.123). A higher maternal BMI was also related to a higher percentage of children’s time spent in sedentary activity (*r* = 0.120, *p* = 0.041) and a lower percentage of children’s time spent in moderate physical activity (*r* = −0.154, *p* = 0.008). No correlation between paternal BMI and physical activity in children was found.

**Table 3:** Correlations between maternal physical activity and BMI and children’s physical activity, BMI and waist circumference

	Physical activity child						Antropometric measures child			
	n	% Sed	% LPA	% MPA	% VPA	Total	n	BMI Z-score	n	WC Z-score
^a,b^Commuting PA (min/wk)	230	−0.081	0.131*	−0.057	−0.054	0.003	1079	−0.062*	735	−0.013
^b^Total PA (min/wk)	230	−0.001	−0.065	0.132*	0.132*	0.097	1079	0.016	735	0.025
^b^LPA (min/wk)	230	−0.010	−0.048	0.112	0.143*	0.096	1079	−0.021	735	−0.016
^b^MPA (min/wk)	230	0.035	−0.035	0.001	0.004	−0.034	1079	0.056	735	0.060
^b^VPA (min/wk)	230	−0.100	0.078	0.063	−0.013	0.064	1079	−0.021	735	−0.032
^c^BMI (kg/m^2^)	291	0.120*	−0.066	−0.154**	−0.109	−0.158**	1448	0.270***	1088	0.215***

*ACM* activity counts per minute, *LPA* light physical activity, *min* minutes, *MPA* moderate physical activity; activity, *PA* physical activity, *Sed* sedentary, *VPA* vigorous physical activity, *WC* waist circumference, *wk* week

*p < 0.05, **p < 0.01, ***p < 0.001

^a^by bike or walking

^b^Spearman correlation coefficient

^c^Pearson correlation coefficient

**Table 4:** Correlations between paternal physical activity and BMI and children’s physical activity, BMI and waist circumference

	Physical activity child						Antropometric measures child			
	n	% Sed	% LPA	% MPA	% VPA	Total	n	BMI Z-score	n	WC Z-score
^a,b^Commuting PA (min/wk)	227	−0.049	0.060	0.066	−0.043	0.016	1023	0.030	688	0.007
^b^Total PA (min/wk)	227	0.031	−0.096	0.075	0.0127	0.044	1023	0.039	688	0.026
^b^LPA (min/wk)	227	−0.027	0.001	0.008	0.006	0.011	1023	0.006	688	0.044
^b^MPA (min/wk)	227	0.104	−0.133*	−0.003	0.098	−0.014	1023	0.031	688	−0.001
^b^VPA (min/wk)	227	−0.034	−0.003	0.085	0.037	0.041	1023	0.015	688	0.015
^c^BMI (kg/m^2^)	291	0.070	−0.052	−0.059	−0.045	−0.067	1405	0.237***	1060	0.203***

*ACM* activity counts per minute, *LPA* light physical activity, *min* minutes, *MPA* moderate physical activity; activity, *PA* physical activity, *Sed* sedentary, *VPA* vigorous physical activity, *WC* waist circumference, *wk* week

*p < 0.05, **p < 0.01, ***p < 0.001

^a^by bike or walking

^b^Spearman correlation coefficient

^c^Pearson correlation coefficient

For both father and mother, total physical activity level was not associated with total physical activity levels of the children, although higher levels of total physical activity in mothers seemed to be associated to more time spent in higher intensity activities in children (moderate physical activity ρ = 0.132, *p* = 0.046; vigorous physical activity ρ = 0.132, *p* = 0.046). In mothers, commuting to school or work either by bike or walking showed a negative correlation with children’s BMI Z-score (ρ = −0.062, *p* = 0.042). Mothers who were active commuters had higher levels of vigorous physical activity than non-active commuting mothers (60 ± 121 vs. 77 ± 131 min per week, *p* = 0.02). The total, light, moderate or vigorous physical activity of the mother or father showed no correlation with the BMI Z-score or waist circumference Z-scores of the children. Within mothers, a higher BMI was associated with lower levels of sports activity, (ρ = −0.085, *p* = 0.003), lower levels of leisure time activity (ρ = −0.083, *p* = 0.003), and lower levels of vigorous physical activity (ρ = −0.084, *p* = 0.003). Within fathers, a higher BMI was associated with more leisure time activity (ρ = 0.075, *p* = 0.011) and lower levels of household activity (ρ = −0.108, *p* < 0.001).

Finally, in children higher levels of total physical activity were related to a higher BMI Z-score (*r* = 0.147, *p* = 0.042, *n* = 193), mainly due to higher levels of moderate and vigorous activities (*r* = 0.149, *p* = 0.039; and *r* = 0.159, *p* = 0.027, respectively; *n* = 193). No significant association between total physical activity and waist circumference Z-score was found (*r* = 0.143, *p* = 0.072, *n* = 159), although higher levels of moderate and vigorous physical activity, and lower levels of light physical activity were significantly related to a higher waist circumference Z-score (*r* = 0.191, *p* = 0.016 and *r* = 0.262, *p* = 0.001, and *r* = −0.173, *p* = 0.029, respectively; *n* = 159).

## Discussion

A higher maternal BMI was associated with a lower total and moderate physical activity level and a higher sedentary activity level of the child. A higher maternal BMI was also related to a higher BMI and higher waist circumference in the child. A higher paternal BMI was most strongly related to a higher BMI and waist circumference in the child. A higher level of maternal total physical activity was related to higher levels of children’s moderate and vigorous physical activity. Also an inverse relationship between active commuting of the mother with children’s BMI was found. Paternal physical activity was not related to children’s physical activity, BMI or waist circumference.

Genetic predisposition, but maybe more important, the obesogenic environment may be underlying factors for these findings. In young children, parents have a strong influence on their children’s lifestyle. They play an important role in the development of habits related to diet and physical activity [13]. Obesogenic behavior in parents is related to obesogenic behavior of the child [22] which causes a higher risk of development of overweight in these children.

Higher levels of maternal active commuting were related to a lower BMI and higher levels of light physical activity in children. We assume that when a mother goes to her work walking or by bike, she will bring her child walking or biking with her to go to school or daycare. These results suggest that when mothers actively participate with their children in daily physical activity, it may contribute to a healthier BMI of their children.

High parental BMI might be an indicator of an obesogenic environment for the child. Indeed, we found that a higher parental BMI is associated to a higher BMI and waist circumference of the child. These finding are in line with the conclusion from a previous review [23]. A higher maternal BMI was also related to lower levels of total, moderate and vigorous physical activity and to higher levels of sedentary activity of the child. We assume that mothers with a higher BMI may create a more obesogenic environment like being less engaged in physical activities together with the child, or less stimulation of physical activity in the child. This fits with our finding that mothers with a higher BMI were less active themselves. In our study, the relation between parental BMI and physical activity in children is attributed to mothers, but not to fathers. In the Netherlands it is common that both mother and father have a job and combine the care for their child, but still mothers spent the most time in raising their child. Women have more often part time jobs compared to men [24], and children of divorced parents live more often with their mother than with their father [25]. We hypothesize that the maternal contribution to an obesogenic environment is stronger than that of the father in preschool age.

Some previous studies investigated the relation between parental physical activity with preschool children’s physical activity, but only few studies measured physical activity with accurate methods, like accelerometry or validated questionnaires [26–29]. Three of the four studies found a positive relationship between parental physical activity and their preschool children’s physical activity. In these studies, children’s physical activity was measured with an accelerometer [26–28] and parental physical activity was measured with an accelerometer [26, 27] or a validated questionnaire [28]. These studies measured physical activity in children for a longer period than that we did in our study, i.e. 5–9 days instead of 4 days. Two studies measured parental physical activity by accelerometer. This might provide more accurate estimates of total parental physical activity than questionnaires. On the other hand, the questionnaire provides more information on type of activity and on structured activities like active commuting. One of the four studies found no direct relation between parental and children’s physical activity, but the previous reported associations may have been indirect, showing that parental physical activity was positively associated with parental physical activity support, which in turn was positively associated with their children’s physical activity [29]. A difference with our study is that their results were based on a physical activity questionnaire for children about physical activity at home instead of a full day physical activity measurement with an accelerometer. They did measure physical activity with an accelerometer, but only while attending child care. For this measurement they found no relation with parental physical activity at all. One study found that the BMI of the father, but not of the mother predicted children’s preschool children’s physical activity level [30]. We found a relation only in mothers, but not in fathers. A difference with our study is that the previous mentioned study measured physical activity of the children while attending daycare, which may be different from physical activity levels at home. Another study did not find a relation between parental BMI and children’s physical activity [27], but they only included 21 fathers and 72 mothers. More research is needed to draw firm conclusions about this relation in preschool children.

We found no studies investigating the relation between parental physical activity and preschool children’s BMI. Studies in older children (7–12 years) and adolescents (11–18 years) did not show consistent results. More research is needed, especially in preschool children.

Some findings were not in line with our hypothesis. We found that more active children had a higher BMI. Surprisingly, this is also found in some previous studies in preschool children [31, 32]. This finding can be explained in various ways. Our finding could be related to the unreliability of BMI as specific indicator of increased fat mass [33, 34]. In some individuals, BMI may also be higher due to fat free mass, in large part muscle mass. This fits with the finding that the association between total physical activity and BMI seems to be determined mostly by the association between high BMI and more moderate/vigorous activity, as vigorous activities are most anabolic and stimulate fitness level and muscle development. It also seems that the relation between physical activity and BMI or body fat can be dependent on age. A review of Jimenez-Pavon et al. [35] found that the relation between physical activity and adiposity was found more often with increasing age. The inverse relation was found in 60 % (*n* = 3) of preschool studies; 77 % (*n* = 17) of studies of primary school-age children, and 86 % (*n* = 18) of studies of adolescents [35]. This last reason is also in line with our finding that waist circumference was not related to total physical activity. We have no specific reason why higher levels of moderate and vigorous physical activity, and lower levels of light physical activity were significantly related to a higher waist circumference.

A strength of our study is the measurement of physical activity of both parents and children with validated instruments and in children with an objective method, the accelerometer. A drawback is that non-validated cut-off points were used to categorize children’s total physical activity into sedentary, light, moderate and vigorous physical activity. The intensity data results may not be generalizable, because our data were used to generate the cut points. Although it can be safely assumed that the activities classified as vigorous are in the high intensity range, the correct classification of in particular the light and moderate activities is less certain. We would advise the validation of the cut points for future research.

## Conclusions

To conclude, BMI of both parents is related to children’s BMI and waist circumference. Higher maternal BMI was related to lower levels of children’s physical activity levels and to higher levels of sedentary activity. Higher maternal physical activity levels were related to lower levels of children’s physical activity. More active commuting by the mother was related to a lower BMI of the children. We suggest that interventions designed to treat or prevent obesity in children should not only focus on the child, but also on the obesogenic environment. Energy-balance related behavior of the parents may contribute to a healthier BMI of both preschool children and their parents.

## Abbreviations

ACM: Activity counts per minute
BMI: Body mass index
